# IL-22 Is Deleterious along with IL-17 in Allergic Asthma but Is Not Detrimental in the Comorbidity Asthma and Acute Pneumonia

**DOI:** 10.3390/ijms241310418

**Published:** 2023-06-21

**Authors:** Amanda Goulart, Mèdéton Mahoussi Michaël Boko, Nubia Sabrina Martins, Ana Flávia Gembre, Rômulo Silva de Oliveira, Sandra Patrícia Palma-Albornoz, Thais Bertolini, Paulo Eduardo Martins Ribolla, Leandra Naira Zambelli Ramalho, Thais Fernanda de Campos Fraga-Silva, Vânia Luiza Deperon Bonato

**Affiliations:** 1 Basic and Applied Immunology Program, Ribeirao Preto Medical School, University of Sao Paulo, Ribeirao Preto 14049-900, Sao Paulo, Brazil; amandagoulart@usp.br (A.G.); bokmich@usp.br (M.M.M.B.); nsmartins@usp.br (N.S.M.); deoliveira.romulo@gmail.com (R.S.d.O.); sandrapalma3@gmail.com (S.P.P.-A.); thabertolini@gmail.com (T.B.); 2 Department of Biochemistry and Immunology, Ribeirao Preto Medical School, University of Sao Paulo, Ribeirao Preto 14049-900, Sao Paulo, Brazil; flagembre@yahoo.com.br (A.F.G.); thaisfragasilva@gmail.com (T.F.d.C.F.-S.); 3 Biotechnology Institute, Sao Paulo State University, Botucatu 18607-440, Sao Paulo, Brazil; p.ribolla@unesp.br; 4 Department of Pathology and Legal Medicine, Ribeirao Preto Medical School, University of Sao Paulo, Ribeirao Preto 14049-900, Sao Paulo, Brazil; lramalho@fmrp.usp.br

**Keywords:** asthma, IL-22, IL-17, eosinophil, dendritic cell, apoptosis

## Abstract

There is evidence that IL-22 and IL-17 participate in the pathogenesis of allergic asthma. To investigate the role of IL-22, we used IL-22 deficient mice (IL-22 KO) sensitized and challenged with ovalbumin (OVA) and compared with wild type (WT) animals exposed to OVA. IL-22 KO animals exposed to OVA showed a decreased number and frequency of eosinophils, IL-5 and IL-13 in the airways, reduced mucus production and pulmonary inflammation. In addition, IL-22 KO animals exhibited a decreased percentage and number of lung CD11c^+^CD11b^+^ cells and increased apoptosis of eosinophils. Th17 cell transfer generated from IL-22 KO to animals previously sensitized and challenged with OVA caused a reduction in eosinophil frequency and number in the airways compared to animals transferred with Th17 cells generated from WT mice. Therefore, IL-22 is deleterious with concomitant secretion of IL-17. Our findings show a pro-inflammatory role for IL-22, confirmed in a model of allergen-free and allergen-specific immunotherapy. Moreover, during the comorbidity asthma and pneumonia that induces neutrophil inflammation, IL-22 was not detrimental. Our results show that targeting IL-22 would negatively affect the survival of eosinophils, reduce the expansion or migration of CD11c^+^CD11b^+^ cells, and negatively regulate allergic asthma.

## 1. Introduction

Asthma affects approximately three hundred million people worldwide [1]. The disease is characterized by bronchial hyperresponsiveness and airflow limitation, caused by airway inflammation [2]. There are different asthma phenotypes. Allergic asthma is characterized by type 2 inflammation, which follows with Th2 cell activation and production of IL-4, IL-5 and IL-13 that cause eosinophilia, pulmonary eosinophilic inflammation, and mucus overproduction, leading to airway dysfunction [3,4]. These characteristics causes coughing, wheezing, and pressure in chest [1]. Severe asthma or type 2 low is characterized by pulmonary neutrophilic inflammation or granulocytic inflammation, Th17 activation and, generally, resistance to corticosteroid treatment [4,5,6].

Studies have shown that Th17 cells may accentuate the severity of the disease, especially in patients who have an intense neutrophil infiltrate in the lungs [4,7]. IL-17A secreted by Th17 cells was detected in samples of bronchial epithelial tissue from patients with more severe symptoms of asthma [8,9]. IL-22, another cytokine of Th17 cells, was also elevated in the serum of asthmatic patients compared to healthy individuals [7,10]. IL-22 acts on epithelial cells, enhances lung tissue integrity and mediates defense against airway infections [11,12,13,14,15]. IL-22 is critical in maintaining the intestinal barrier because it induces the expression of anti-apoptotic proteins responsible for the survival of epithelial cells in the intestine [16]. Moreover, IL-22 production might be regulated by intestinal bacteria [17] and IL-22 modulates intestinal microbiota [18,19].

IL-22 might play a pro- or anti-inflammatory role depending on the microenvironment [10,20,21]. In the airway inflammation after allergen exposure, IL-22 functions are still controversial. The neutralization of IL-22 positively modulated eosinophil recruitment to the lungs in an ovalbumin (OVA)-induced asthma model [22]. Pretreatment of OVA-pulsed dendritic cells (DC) with recombinant IL-22 abolished the eosinophil recruitment induced by those non-treated and OVA-pulsed DC [22]. Administration of anti-IL-22 monoclonal antibody augmented eosinophil recruitment and IL-13 secretion while recombinant IL-22 reduced eosinophilic inflammation and indirectly decreased the secretion of IL-25 by airway epithelial cells [23]. These studies showed that IL-22 acts as a negative regulator of type 2 inflammation in the airways. The pro- or anti-inflammatory action of IL-22 was described to be time-dependent given that the neutralization of this cytokine during the sensitization with allergen or sensitization of IL-22 deficient mice ameliorated and reduced allergic inflammation, and decreased eosinophilic inflammation while recombinant IL-22 given during the allergen challenge was protective [10].

In an attempt to investigate the role of IL-22 in asthma, which is still controversial, we use IL-22 knockout mice to evaluate its role on airway inflammation, eosinophil survival and number of dendritic cells.

## 2. Results

### 2.1. IL-22 Has a Pro-Inflammatory Function in Airway Allergic Inflammation

**Figure 1:**
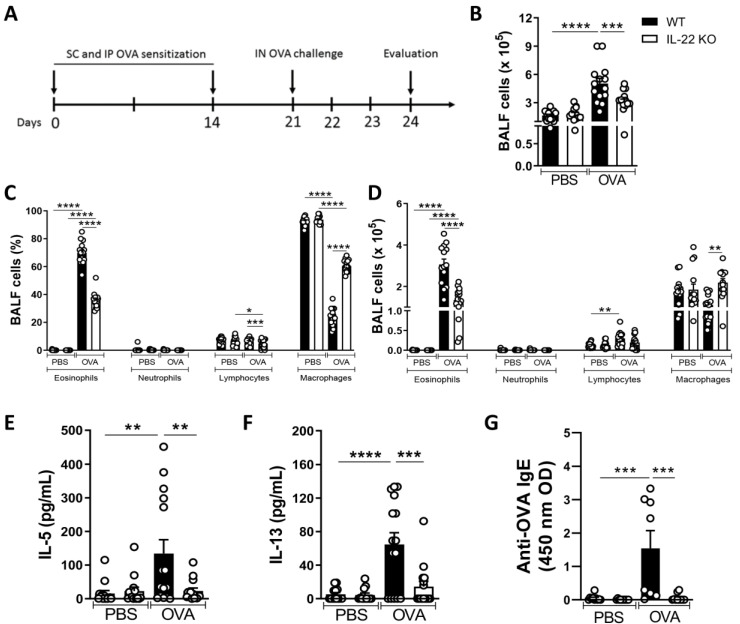
**The allergic response is improved in the absence of IL-22.** (**A**) WT and IL-22 KO mice were sensitized by administration of OVA (100 μg) and Aluminum Hydroxide (1.6 mg) by subcutaneous route (SC). On day 14, the animals received the second OVA sensitization (50 μg) via intraperitoneal route (IP). On day 21, the animals were challenged with OVA (100 μg) via the intranasal route (IN). (**B**) Total number of cells in BALF. (**C**,**D**) Differential and total count of BALF leukocytes. (**E**,**F**) Concentrations of IL-5 and IL-13 in the BALF supernatants. (**G**) Concentrations of anti-OVA IgE in the serum. The results are expressed as mean ± standard deviation of the individual values obtained for each experimental group. Results are representative of three independent experiments for BALF and cytokines and two independent experiments for antibodies. * *p* < 0.05; ** *p* < 0.01; *** *p* < 0.001; **** *p* < 0.0001. IP = intraperitoneal route, SC = Subcutaneous, IN = Intranasal.

**Figure 2:**
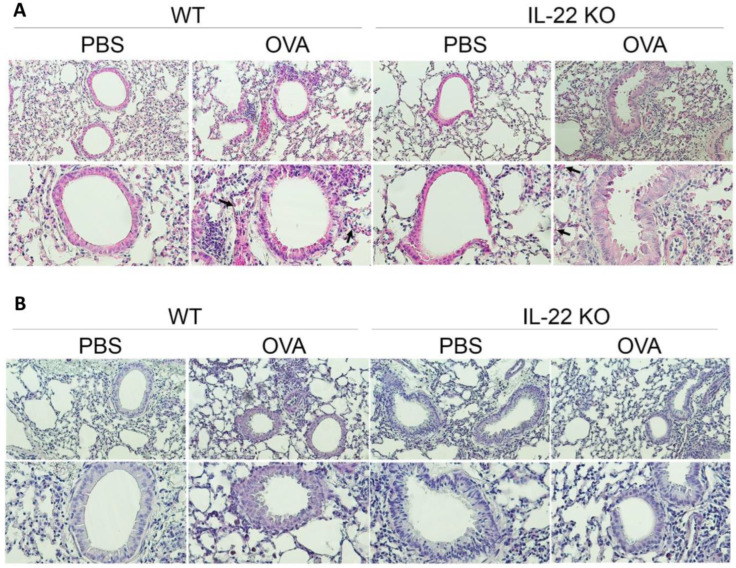
**Reduction in pulmonary inflammation and mucus production in the absence of IL-22.** (**A**) Histopathological analysis of the lungs. Arrows show eosinophils. (**B**) Mucus production. Results are representative of three independent experiments. Top = magnification 200×; bottom = magnification 400×.

To confirm the pro- or anti-inflammatory function of IL-22 in the airway allergic inflammation, WT and IL-22 KO mice sensitized and challenged with OVA had their lungs evaluated, as depicted in Figure 1A. The total number of cells in the BALF was quantified and showed a significant decrease in numbers in the BALF of IL-22 KO mice exposed to OVA (IL-22 KO OVA) compared to their counterpart WT (WT OVA) (Figure 1B). The frequency and the number of eosinophils and lymphocytes were also reduced in the BALF of the IL-22 KO OVA group compared to WT OVA animals (Figure 1C,D). There was no difference in the number of neutrophils, as they were too low in all groups, and macrophages among the groups (Figure 1C,D). Considering the decrease in BALF cells in IL-22 KO OVA animals, we evaluated the production of Th2 cytokines. Corroborating the data of lymphocytes, concentrations of IL-5 and IL-13 were also reduced in the BALF of IL-22 deficient mice exposed to the allergen (Figure 1E,F). In addition, the IL-22 KO OVA group exhibited reduced levels of circulating OVA-specific IgE compared to those detected in the WT OVA group (Figure 1G). Histological analysis of WT OVA group revealed increased inflammatory cells, including eosinophils (arrows) (Figure 2A-top panel). The inflammatory infiltrate was predominantly peribronchial (Figure 2A-bottom panel). WT animals exposed to OVA exhibited hyperplasia of goblet cells and mucus production (Figure 2B). IL-22 KO OVA group exhibited mild pulmonary inflammation (Figure 2A) and no mucus production (Figure 2B) compared to the respective WT group. However, the number of inflammatory cells in IL-22 deficient animals sensitized and challenged with OVA were increased compared to IL-22 deficient animals non-exposed to the allergen (Figure 2A).

These results attribute a pro-inflammatory function to IL-22 in the allergic airway inflammation given that airway allergic type 2 inflammation was impaired in IL-22 deficient mice.

### 2.2. Increase in IL-17 in the Lungs of Animals Exposed to the Allergen Is Not Affected by the Deficiency of IL-22

**Figure 3:**
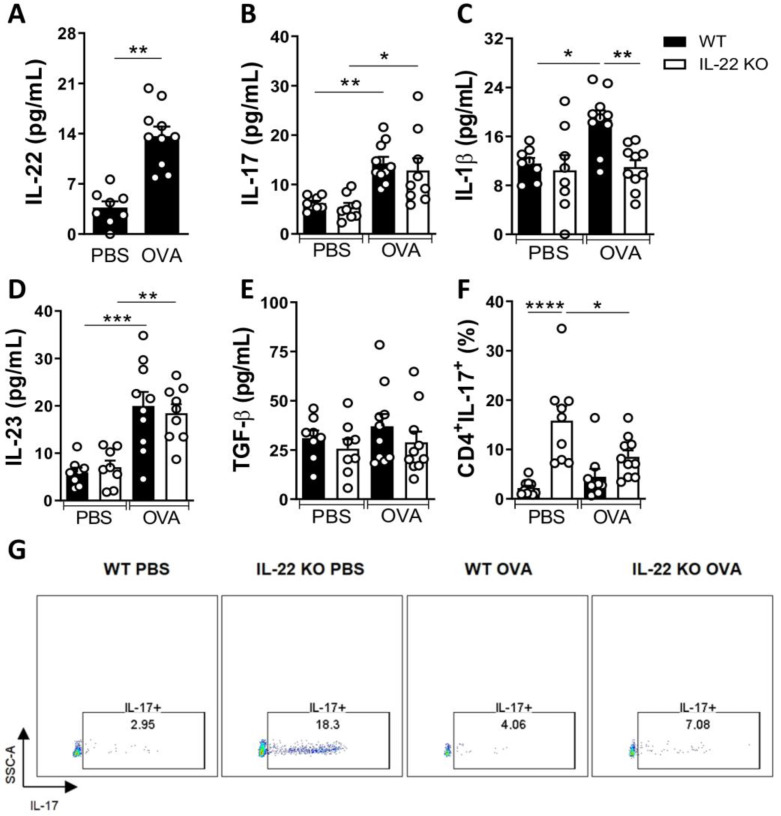
**Th17 response is induced in allergic lung inflammation.** (**A**,**B**) Concentrations of IL-22 and IL-17 in the BALF supernatants. (**C**–**E**) Concentrations of IL-1β, IL-23 and TGF-β in the BALF supernatants. (**F**,**G**) Frequency and representative analysis of CD4^+^IL-17^+^ cells, gated previously on CD4^+^ cells, in the lungs of mice exposed or not (PBS) to the allergen (OVA). Results were expressed as mean ± standard deviation of the individual values obtained for each experimental group. Results are representative of two independent experiments. * *p* < 0.05; ** *p* < 0.01; *** *p* < 0.001; **** *p* < 0.0001.

IL-17 and IL-22 are increased in the lungs and in the serum of asthmatics subjects compared to healthy subjects [7,10]. We evaluated the production of IL-17 in immune competent and in IL-22 deficient-mice exposed to the allergen. WT mice exposed to the allergen (WT OVA group) showed an increase in IL-22 and IL-17 concentrations in the BALF compared to the control group (WT PBS). IL-22 KO mice exposed to the allergen (IL-22 KO OVA) also secreted significant concentrations of IL-17 compared to their control counterpart (IL-22 KO PBS) (Figure 3A,B).

IL-1β, described as an important cytokine in the Th17 cell differentiation [24], was enhanced in WT OVA group, but not in the IL-22 KO OVA group (Figure 3C). In addition, IL-23, which maintains the Th17 differentiation [25], was enhanced in both WT and IL-22 KO mice exposed to the allergen (Figure 3D). TGF-β concentrations were not altered among experimental groups (Figure 3E).

Next, we evaluated the frequency of IL-17-producing CD4^+^ cells in the lungs and we found no difference between WT OVA and IL-22 KO OVA groups. However, the deficiency of IL-22 increased the frequency of IL-17-producing CD4^+^ cells in the control group (PBS) compared to the WT PBS group (Figure 3F,G). Although the deficiency of IL-22 increases the frequency of IL-17-producing CD4^+^ cells in steady-state (IL-22 KO PBS group), in the presence of allergens, IL-22 KO animals (IL-22 KO OVA group) exhibited lower frequency of IL-17-producing CD4^+^ cells (Figure 3F,G).

### 2.3. Th17 Cells Negatively Regulate Eosinophil Recruitment in the Absence of IL-22

**Figure 4:**
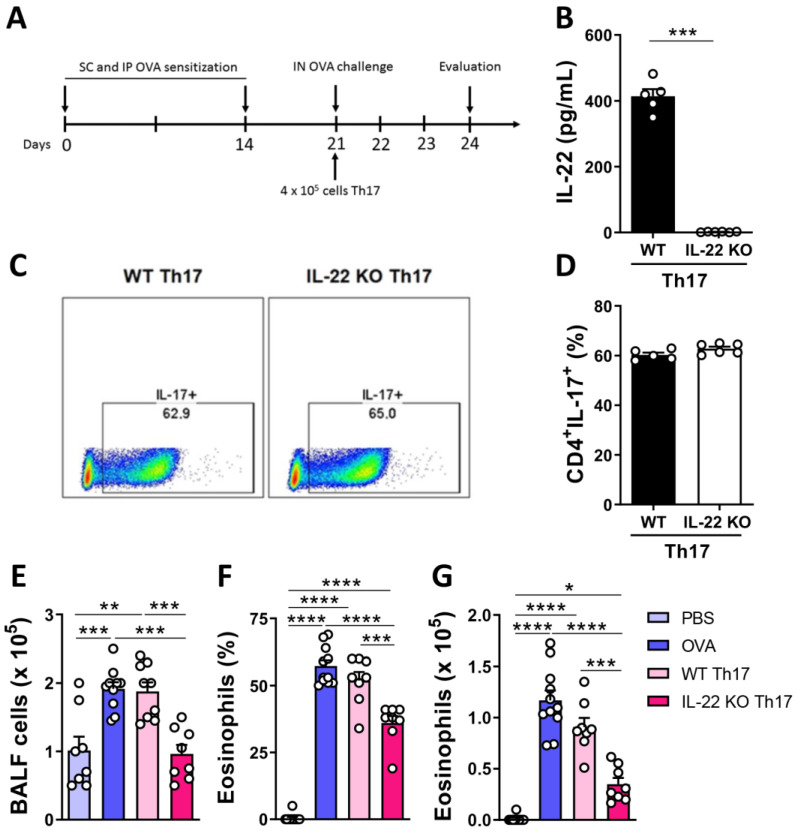
**In the absence of IL-22, Th17 cells are protective in allergic asthma.** (**A**) WT animals were sensitized and challenged, as previously described, and during the challenge, they received 4 × 10^5^ Th17 cells differentiated from naive CD4^+^CD62L^+^ cells from the spleen of WT or IL-22 KO animals. (**B**) Concentrations of IL-22 secreted by differentiated Th17 cells in vitro. (**C**,**D**) Representative flow cytometry analysis and frequency of differentiated Th17 cells in vitro. (**E**) Total number of cells in the BALF. (**F**,**G**) Frequency and total number of eosinophils in the BALF. Results are expressed as mean ± standard deviation of the individual values obtained for each experimental group. Results are representative of two independent experiments. * *p* < 0.05; ** *p* < 0.01; *** *p* < 0.001; **** *p* < 0.0001. IP = intraperitoneal route, SC = Subcutaneous, IN = Intranasal.

Neutralization of IL-17 in IL-22 KO mice exposed to the allergen restores eosinophil recruitment in the lungs [10]. In an attempt to confirm that the concomitant secretion of IL-17 and IL-22 by Th17 cells play a pro-inflammatory role, we differentiated Th17 cells in vitro from naive CD4^+^ lymphocytes obtained from spleens of WT or IL-22 KO mice and performed Th17 cell transfer at the moment of challenge with OVA (Figure 4A). First, we confirmed that Th17 cells differentiated from WT animals produced both IL-17 and IL-22 (Figure 4B–D). As expected, Th17 cells from IL-22 KO animals produced only IL-17 (Figure 4B–D). Th17 cell transfer from WT mice did not significantly affect total cell number, frequency and number of eosinophils in the BALF compared to mice sensitized and challenged with OVA with no cell transfer (Figure 4E–G). However, Th17 cell transfer from IL-22 KO mice to mice sensitized and challenged with OVA significantly reduced total cell number, frequency and number of eosinophils in the BALF (Figure 4E–G). These findings show that IL-17 and IL-22 act concomitantly to induce a type 2 inflammation in allergic asthma.

### 2.4. IL-22 Increases the Frequency of Lung CD11c^+^CD11b^+^ Dendritic Cells and Promotes Viable Eosinophils

**Figure 5:**
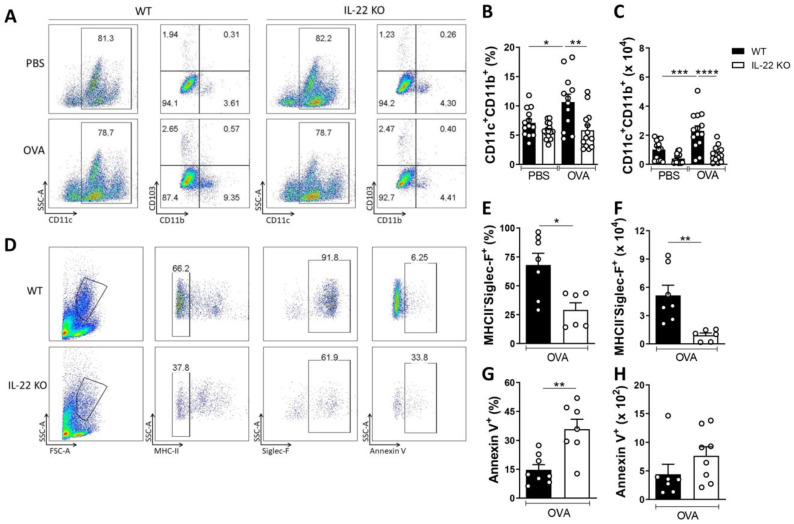
**IL-22 modulates the frequency and number of dendritic cells, and eosinophil apoptosis in lungs of mice exposed to the allergen.** WT and IL-22 KO mice were sensitized and challenged with OVA, as described in the legend of Figure 1A. (**A**) Representative flow cytometry analysis of CD11c^+^CD11b^+^CD103^−^ cells in the lungs. (**B**,**C**) Frequency and number of CD11c^+^CD11b^+^CD103^−^ cells in the lungs. (**D**) Representative flow cytometry analysis of MHC-II^−^Siglec-F^+^ cells and MHC-II^−^Siglec-F^+^AnnexinV^+^ cells. (**E**,**F**) Frequency and number of MHC-II^−^Siglec-F^+^ cells. (**G**,**H**) Frequency and number of MHC-II^−^Siglec-F^+^AnnexinV^+^ cells. The results are expressed as mean ± standard deviation of the individual values obtained for each experimental group. Results are representative of three independent experiments for DC and two independent experiments for eosinophil apoptosis. * *p* < 0.05; ** *p* < 0.01; *** *p* < 0.001; **** *p* < 0.0001.

IL-22-treated and OVA-pulsed DC impaired the recruitment of eosinophils to the airways [22]. Given that IL-22 affects the role of dendritic cells, we asked whether IL-22 plays a role in the DC infiltration of the airways following allergen exposure. We found a decrease in the frequency and in the number of CD11c^+^CD11b^+^ DC, which is the DC subset that orchestrates Th2 immune response in asthma [26,27], in the lungs of IL-22 deficient mice exposed to OVA compared to the WT OVA group (Figure 5A–C). No differences were observed in the frequency of CD11c^+^CD103^+^ DC.

IL-22 also participates in the induction of anti-apoptotic factors [28,29]. Considering the roles of IL-22 and that IL-22 may also promote the in vitro survival of epithelial cell [11,16], we asked whether IL-22 would promote eosinophil survival. Pulmonary eosinophils were evaluated as MHC class II- and Siglec-F^+^ cells [30]. Our results show that IL-22 deficient mice exhibited a percentage and number of eosinophils lower than the WT OVA group (Figure 5D–F). We found that in a lower frequency and number the population of eosinophils in the lungs of IL-22 KO animals exhibited greater annexin V staining, confirming that the absence of IL-22 causes an increase in apoptosis in eosinophils (Figure 5D,G,H).

These results suggest that the pro-inflammatory function of IL-22 is associated with the recruitment and/or expansion of CD11b^+^ DC that favor Th2 immune response and that IL-22 prevents eosinophil apoptosis.

### 2.5. Allergen-Specific and Allergen-Free Treatment Reduces Eosinophil, Dendritic Cell and IL-22 Secretion in Airway Lung Inflammation

**Figure 6:**
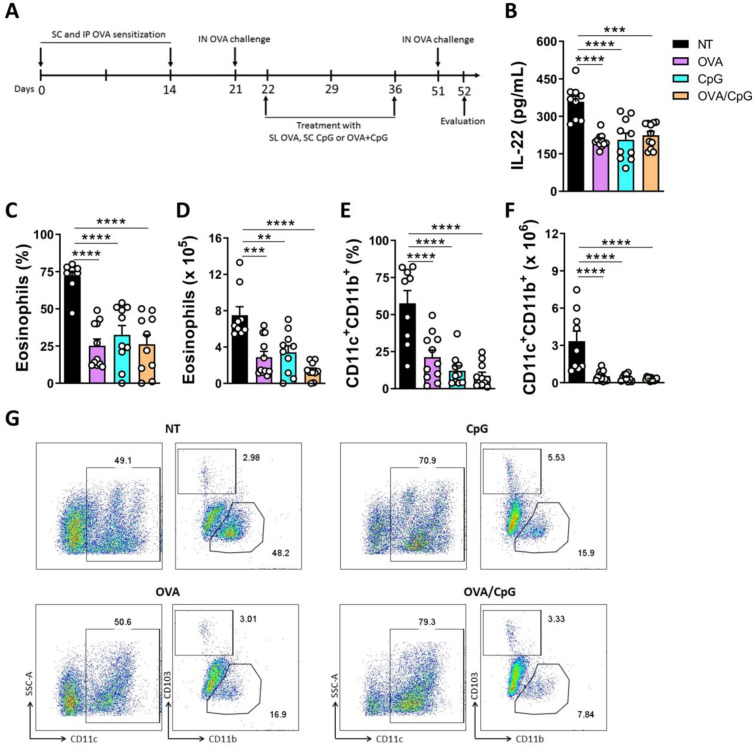
**Allergen-specific, allergen-free or simultaneous treatment reduces IL-22 production and dendritic cells in the lungs.** (**A**) Mice were sensitized and challenged with OVA, followed by treatment with OVA by sublingual route, or CpG by subcutaneous route or with OVA and CpG simultaneously. Fifteen days after the end of immunotherapy, mice were challenged again with OVA, and evaluated 72 h post challenge. (**B**) Levels of IL-22 in the BALF. (**C**,**D**) Frequency and number of eosinophils in the BALF. (**E**,**F**) Frequency and number of CD11c^+^CD11b^+^ cells in the lungs of treated or non-treated animals. (**G**) Representative flow cytometry analysis of CD11c^+^CD11b^+^ cells in the lungs of treated or non-treated animals. The results are expressed as mean ± standard deviation of the individual values obtained for each experimental group. Results are representative of two independent experiments. The bars show the significant differences between groups ** *p* < 0.02; *** *p* < 0.01; **** *p* < 0.0001. IP = intraperitoneal route, SC = Subcutaneous, IN = Intranasal, SL = Sublingual.

Our group reported previously the mechanisms by which CpG plus mycobacterial protein, an allergen-free immunotherapy, conferred protection against pulmonary eosinophilic inflammation [31,32,33]. To confirm the role of IL-22 as a pro-inflammatory cytokine in the airway lung inflammation, we evaluated if the levels of this cytokine would be reduced after a mixed allergen-specific and allergen-free immunotherapy. WT animals were sensitized and challenged with OVA and treated with increasing concentrations of OVA by sublingual route, or CpG oligodeoxynucleotides, agonist for TLR9, by subcutaneous route, or treated simultaneously with OVA by sublingual route and CpG by subcutaneous route (Figure 6A). Treated animals showed a significant decrease in IL-22 in the lung homogenates (Figure 6B). Concomitantly, treated groups exhibited a significant reduction in the allergic inflammatory response, characterized by decrease in frequency and number of eosinophils in the BALF (Figure 6C,D) associated with the reduction in frequency and number of CD11c^+^CD11b^+^ DC (Figure 6E–G). These findings confirm the role of IL-22 as a pro-inflammatory mediator in lung allergic inflammation.

### 2.6. IL-22 Plays No Role in the Comorbidity Asthma and Acute Pneumonia

**Figure 7:**
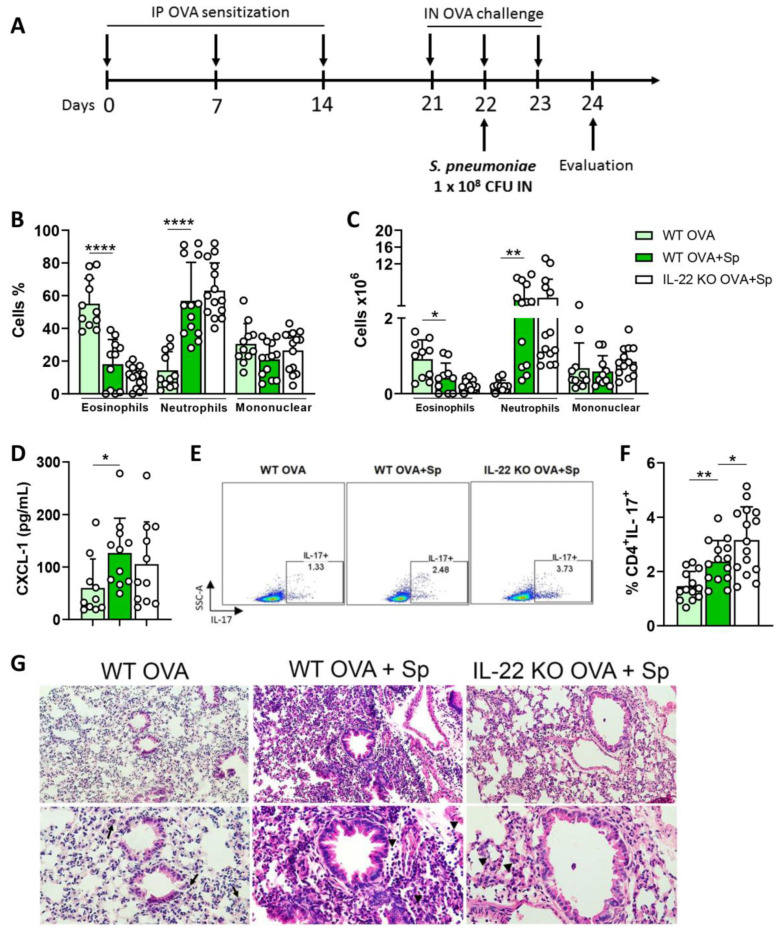
**IL-22 is not required for the exacerbation of neutrophilic inflammation in the comorbidity asthma and acute pneumonia.** (**A**) WT and IL-22 KO animals were sensitized and challenged with OVA and infected or not with *S. pneumoniae*. (**B**,**C**) Differential and total count of leukocytes in the BALF. (**D**) Concentrations of CXCL-1 in the BALF supernatants. (**E**,**F**) Frequency of CD4^+^IL-17^+^ cells in the lungs. (**G**) Histopathological analysis of the lungs. Arrows and arrowheads show eosinophils and neutrophils, respectively. The results are expressed as mean ± standard deviation. Results are representative of three independent experiments. Top, magnification 200×; bottom, magnification 400×. The bars show the significant differences between groups * *p* < 0.05; ** *p* < 0.01; **** *p* < 0.0001.

Next, we evaluated whether the IL-22 deficiency would affect allergic asthma that courses with neutrophil inflammation. For this, we employed a model of comorbidity of allergic asthma with acute pneumonia caused by *Streptococcus pneumoniae*, as depicted in Figure 7A, because pneumococcus changes the pattern of airway eosinophilic inflammation to a mixed neutrophilic/eosinophilic inflammation. We observed a significant increase in the recruitment of neutrophils accompanied by a reduction in the recruitment of eosinophils in the BALF of WT mice exposed to OVA and infected with *S. pneumoniae* (OVA+Sp group) compared to the animals exposed only to the allergen (WT OVA group) (Figure 7B,C). Additionally, the concentrations of CXCL-1, a neutrophil chemoattractant, the frequency of CD4^+^IL-17^+^ cells and the pulmonary inflammation were increased in the lungs during the comorbidity compared to the WT OVA group (Figure 7D–G). The induction of comorbidity in IL-22 KO group increased the percentage of CD4^+^IL-17^+^ cells in the lung compared to WT counterpart (WT OVA+Sp group) (Figure 7E,F). Neutrophilic inflammation, CXCL-1 levels and pulmonary inflammation were not significantly different when comparing WT OVA+Sp and IL-22 KO OVA+Sp groups (Figure 7B–G), suggesting that IL-22 is not involved in the comorbidity asthma and acute pneumonia that courses with neutrophilic inflammation. These findings suggest that IL-22 plays a pivotal role in allergic asthma but is dispensable for asthma that coursed with an increase in neutrophils.

## 3. Discussion

Studies show that the IL-22 function in airway allergy appears to be time-dependent, whereas IL-22 was protective during the allergen challenge and deleterious during the sensitization [10]. Moreover, the deleterious role of IL-22 was dependent on the simultaneous presence of IL-17 [10]. However, two studies attributed an anti-inflammatory function to IL-22 [22,23], while one study showed a pro-inflammatory role to this cytokine [10]. The findings reported in these studies show that the IL-22 function in the airway allergy remains to be elucidated given that the collecting findings are controversial [10,22].

Our results show a significant increase in the production of IL-22 and IL-17 in the BALF of mice exposed to the allergen, confirming the studies of other groups [7,10]. The deficiency of IL-22 did not affect the frequency of IL-17-producing CD4^+^ cells in the lungs of mice exposed to the allergen. However, mice deficient in IL-22 exhibited a higher frequency of IL-17-producing CD4^+^ cells, as already described in a model of lung inflammation induced by silica [34]. Therefore, IL-22 appears to negatively regulate Th17 cells in steady state. In the silica model, IL-17 is mostly produced by T cells in the lungs, and it is essential for an increase in inflammatory infiltrate in the airways [34]. Our study shows an original finding when we described that the pro-inflammatory function of IL-22 was dependent on simultaneous secretion of IL-17 by Th17 cells in allergic asthma.

The increase in IL-17 and IL-22 occurred along with the significant increase in IL-1β and IL-23 in the BALF. IL-1β and IL-23 participate in the differentiation and maintenance of Th17 cells [35]. As IL-17 and IL-22 are elevated in mice exposed to the allergen, as well as in patients presenting symptoms of severe or moderate asthma, our study and our results suggest that IL-22 plays a pro-inflammatory function in airway allergic inflammation modulating it positively, as described by Besnard et al. [10], because IL-22 deficiency caused a reduction in eosinophils and lymphocytes in the BALF, IL-5 and IL-13 reduced inflammatory cell infiltrate in the airways and decreased mucus production by goblet cells in the lungs compared to WT group exposed to the allergen.

The studies with IL-22 in asthma are scarce and suggest that this cytokine negatively reduces the pro-allergenic role of dendritic cells (DC) [22]. Because we showed a pro-inflammatory function for IL-22 in allergic asthma, we evaluated the frequency and number of CD11c^+^CD11b^+^ DC ex vivo, which were negatively regulated in IL-22 KO mice exposed to the allergen compared to WT OVA group. CD11c^+^CD11b^+^ DC were described as main players in generating Th2 immune response following allergen contact [26,27]. Therefore, we show an ex vivo finding that IL-22 might downregulate the recruitment or expansion of CD11c^+^CD11b^+^ DC, which contribute to the differentiation and proliferation of IL-5- and IL-13-producing effector and memory Th2 cells and eosinophil recruitment. It remains to be evaluated if the treatment with recombinant IL-22 would be capable of increasing the infiltration of CD11c^+^CD11b^+^ DC in the lungs of animals exposed to the allergen.

We also found that the deficiency of IL-22 increased the frequency of eosinophils in apoptosis (MHC-II^−^Siglec-F^+^AnnexinV^+^ cells), suggesting that IL-22 is also a survival factor for eosinophil and not only for epithelial cells [16]. This result suggests that IL-22 may indirectly influence the maintenance of lung allergic inflammation, acting on the survival of the most important readout of experimental allergic inflammation, the eosinophil. It remains to be confirmed in vitro if IL-22 increases the survival of eosinophils.

Previously, our group used allergen-free immunotherapy and showed that CpG plus proteins secreted by *Mycobacterium tuberculosis* [31] or CpG plus mycobacterial protein [33] were capable to negatively regulate allergic asthma, reduce eosinophilic and Th2 inflammation by a mechanism dependent on the deviation of immune response to Th1 cells and by a mechanism dependent on TLR9 and increase the influx of inflammatory monocytes. Moreover, Fonseca et al. [32] showed that mice with pulmonary allergic inflammation treated with CpG exhibited a decrease in IL-17 concentrations in the BALF compared to the group exposed to the allergen but non-treated. This study attributes to IL-17 a pro-inflammatory role in allergy of the airways. However, the role of IL-22 had not yet been assessed [32]. To confirm that IL-22 has a pro-inflammatory function, we treated mice exposed to the allergen with different treatments: allergen-free therapy (CpG treatment), allergen-specific therapy (increasing doses of OVA), and a combination of both CpG and increasing doses of OVA, and evaluated if the immunotherapy would be able to reduce IL-22 concentrations as well as eosinophilic inflammation on the BALF, and influx of CD11c^+^CD11b^+^ cells in the lungs. We used sublingual, specific-allergen immunotherapy because it has been shown to be effective in reducing pulmonary allergic inflammation by promoting peripheral tolerance, regulatory T lymphocytes, and IL-10 production [36,37]. The role of IL-22 in the sublingual allergen-specific immunotherapy had not been elucidated. As the two forms of therapy had already been described in the literature, we sought to assess whether combined allergen-specific therapy and allergen-free therapy could potentiate the reduction in allergic inflammation and IL-22 production. Our results show that the reduction in frequency and number of eosinophils and CD11c^+^CD11b^+^ cells were accompanied by a reduction in IL-22 concentrations, confirming that IL-22 acts as a pro-inflammatory mediator in the airway allergy inflammation.

Although we show that IL-22 positively regulates the allergic asthma inducing eosinophil survival and CD11c^+^CD11b^+^ cell infiltration, IL-22 plays no pro-inflammatory role in neutrophilic inflammation induced during the comorbidity asthma and acute pneumonia. During the comorbidity asthma and acute pneumonia, we found an increase in CD4^+^IL-17^+^ cells in the lungs of WT animals exposed to the allergen and infected with *S. pneumoniae*. However, the deficiency of IL-22 neither affects pulmonary inflammation nor neutrophil or eosinophil frequencies. These results corroborate data from patients with severe asthma that have an increased neutrophilic inflammation and Th17 cells, suggesting that IL-17, and not IL-22, might contribute to asthma severity that courses with neutrophil infiltration [4,7].

Although our study has limitations, as it would have been of great importance to investigate the death by apoptosis of human eosinophils differentiated in vitro or obtained from BALF of asthmatics patients, beyond the frequency of CD11c^+^CD11b^+^ cells in the peripheral blood and in the BALF of asthmatics patients, our study adds new clues about the pro-inflammatory role of IL-22 in allergic asthma.

## 4. Materials and Methods

### 4.1. Mice

Female C57BL/6 wild type (WT) and IL-22-KO mice, 8–12 weeks, were obtained from the local breeding facility at the Ribeirao Preto Medical School, University of Sao Paulo, Brazil, and housed under specific pathogen-free conditions. Mice were treated according to the Animal Welfare guidelines of the Ribeirao Preto Medical School, University of Sao Paulo. The local Animal Research Ethics Committee approved all of the procedures (Process number 140/2014 and 245/2019).

### 4.2. Induction of Allergic Asthma

WT and IL-22-KO mice were sensitized with 100 μg of OVA Grade V (Sigma-Aldrich, St. Louis, MO, USA, A5503) and aluminum hydroxide (1.6 mg) subcutaneously on day 0 and sensitized again with OVA (50 μg) intraperitoneally on day 14. The animals were challenged with OVA (100 μg) via the intranasal route on day 21, and 72 h after the challenge (day 24), the animals were euthanized, and lungs and serum were collected. The euthanasia of the animals was performed by retro-orbital inoculation of 100 μL of anesthetic solution containing 20% ketamine hydrochloride (Agener, Embu-Guaçu, Sao Paulo, Brazil) and 10% xylazine (Laboratorios Calier SA, Barcelona, Spain).

### 4.3. Induction of Th2 Low Asthma

WT and IL-22-KO mice were sensitized three times, at 7-day intervals, with 10 µg of OVA Grade VI (Sigma-Aldrich, St. Louis, MO, USA, A2512) and aluminum hydroxide (2 mg) (Thermo Scientific, Waltham, MA, USA, 77161) intraperitoneally. On days 21, 22 and 23 mice were challenged with 30 µg OVA Grade V (Sigma, A5503), intranasally, and samples were collected 24 h after the last challenge. TIGR4 strain of *S. pneumoniae* (ATCC BAA-334) frozen at −80 °C in tryptic soy broth (TSB) (BD, cat. 211825) containing 10% glycerol was thawed, plated on blood agar and incubated overnight at 37 °C, 5% CO_2_. After growth, colonies were inoculated in TSB medium, when the growth was checked at OD 600 = 0.06, following incubation for 4 to 5 h. When OD 600 = 0.3, bacteria was counted, and the concentration was adjusted to 50 × 10^8^/mL of colony-forming unit (CFU) in sterile Phosphate Buffered Saline (PBS). Mice were infected with 1 × 10^8^ CFU of *S. pneumoniae* by intranasal route (20 µL/animal) before the second challenge with OVA. The euthanasia of the animals was performed 48 h after the infection.

### 4.4. BALF

Mice were anesthetized with ketamine (100 mg/kg, Sespo Industry and Commerce, Paulínia, SP, Brazil) and xylazine (10 mg/kg, Vetecia Laboratory of Veterinary Products, Jacareí, SP, Brazil) 72 h after OVA challenge or 24 h after the last OVA challenge. The trachea was exposed and cannulated with a subsequent injection of 5 mL of RPMI 1640 (Sigma-Aldrich, St. Louis, MO, USA) for bronchoalveolar lavage fluid (BALF) collection. Samples were centrifuged at 450× *g* for 10 min and the supernatants were stored at −20 °C for cytokine measurement. Cells were resuspended in 500 µL of RPMI 1640 and centrifuged at cytocentrifuge (Thermo Fisher Scientific, Waltham, MA, USA) at 18× *g* for 3 min. Next, cells were stained with rapid panoptic (Labor Clin, Sao José do Rio Preto, SP, Brazil) for differential cell count.

### 4.5. Th17 Cell Differentiation

Naive T cells (CD4^+^CD62L^+^) were isolated from total spleen cells of WT or IL-22-KO animals by MACS CD4^+^CD62L^+^ cell isolation kit II (Miltenyi Biotec, Bergisch Gladbach, Germany). Purified 1.5 × 10^6^ cells (CD4^+^CD62L^+^) were distributed in 96-well plates (Greiner Bio-one, Kremsmünster, Austria) in IMDM medium (Sigma, St. Louis, MO, USA) (Sigma, St. Louis, MO, USA), Sodium Pyruvate (400 μM) (Sigma, St. Louis, MO, USA), L-glutamine (0.88 μM) (Sigma-Aldrich, St. Louis, MO, USA), 2-Mercaptoethanol (55 μM) (Sigma, St. Louis, MO, USA) and gentamycin (10 μg/mL) (Gibco-Invitrogen, Grand Island, NY, USA). For the differentiation of Th17 cells, naive cells were cultured with antibodies required for polyclonal stimulation, mAb anti-CD3 (2 μg/mL) (BD Biosciences, Franklin Lakes, NJ, USA) and mAb anti-CD28 (2 μg/mL) (BD Biosciences, Franklin Lakes, NJ, USA), recombinant cytokines for Th17 polarization: IL-6 (20 ng/mL) (R&D Systems, Minneapolis, MN, USA), TGF-β (5 ηg/mL) (R&D Systems, Minneapolis, Minnesota), in addition to mAb anti-IL-4 (20 μg/mL) (R&D Systems, Minneapolis, MN, USA), mAb anti-IL-2 (20 μg/mL) (R & D Systems, Minneapolis, MN, USA) and mAb anti-IFN-γ (20 μg/mL) (R&D Systems, Minneapolis, MN, USA). After 72 h of culture, the supernatants were collected for detection of IL-17 and the cells were analyzed by flow cytometry for quantification of CD4+ IL-17+ cells. Th17 cell transfer (4 × 10^5^ cells) was performed via the intra-tracheal route.

### 4.6. Lung Cells

After euthanasia, lungs were perfused with 5 mL of PBS through the right ventricle. Perfused lower right lobes lungs were cut into small pieces and digested for 50 min at 37 °C, 5% CO_2_ with collagenase type IV (2.2 mg/mL) (Sigma-Aldrich, St. Louis, MO, USA) and DNAse (0.055 mg/mL) (Roche, Basel, Switzerland). The digestion was stopped using EDTA (10 mM). Then, cells suspension was passed through a cell strainer, centrifuged, lysed with ACK, and resuspended in complete RPMI 1640 (Sigma-Aldrich, St. Louis, MO, USA).

### 4.7. Flow Cytometry

Isolated lung cells were stained with FVS780 viability dye (BD Pharmingen, San Diego, CA, USA) and specific antibodies for the characterization of Th17 cells (CD4^+^IL-17^+^), dendritic cells (CD11c^+^CD11b^+^CD103^−^) and eosinophils (MHCII^+^Siglec-F^+^) were used according to manufacturer’s instructions (BD Pharmingen, San Diego, CA, USA and Thermo Fisher Scientific, Waltham, MA, USA). To evaluate eosinophils death, lung cells were stained with Annexin V (BD Pharmingen, San Diego, CA, USA) after antibodies staining. Samples were fixed with paraformaldehyde 1% (Labsynth, Diadema, SP, Brazil). Samples were acquired in FACS Canto (BD Biosciences, San Jose, CA, USA) and analyses were performed in the FlowJo X 10.0.7r2 software for Windows (Becton Dickinson and Company, Franklin Lakes, NJ, USA).

### 4.8. IgE Levels

OVA-specific IgE antibodies were assayed in the mouse serum by sandwich ELISA using biotin-conjugated anti-mouse IgE (Clone R35-118, BD Pharmingen, San Diego, CA, USA). First, a 96-well plate was coated with OVA Grade V (Sigma, A5503) and incubated overnight at 4 °C. After washing, nonspecific binding was blocked with PBS-10% fetal bovine serum and 0.05% Tween 20 during 1 h at 37 °C. The plate was washed again, and serum samples were added and incubated for 2 h at 37 °C. Biotin-conjugated anti-mouse IgE was added after washing and the plate was incubated for 1 h at 37 °C. The reaction was revealed using tetramethylbenzidine substrate (BD Pharmingen, San Diego, CA, USA) and stopped with 16% sulfuric acid solution. The results were expressed as absolute absorbance values measured at 450 nm.

### 4.9. Pulmonary Inflammation and Mucus Production Histological Analysis

The upper right lobe of the lung was preserved in buffered formalin for the preparation of tissue slides. Pulmonary tissue samples were cut (5 μm) and stained with hematoxylin-eosin (HE). The analysis of mucus production was performed by periodic acid staining of Schiff (PAS).

### 4.10. Statistical Analysis

All data were initially analyzed for normal/parametric distribution (Kolmogorov–Smirnov test). If there was a parametric distribution, the analysis of variance was applied to evaluate the differences among groups. To compare 2 groups, Student’s T-test was applied. If no parametric distribution was found, the Kruskal–Wallis test was applied to evaluate differences among groups. To compare 2 groups, the Mann–Whitney U test was applied. Statistical analyzes were performed using GraphPad PrismVersion 8.1 (GraphPad 8.0.2 software for Windows, Inc., San Diego, CA, USA). Values of *p* < 0.05 were considered significant.

## 5. Conclusions

In summary, our data suggest that IL-22 acts as a pro-inflammatory cytokine in allergic asthma, contributing to the survival of eosinophils and increase in CD11b^+^ DC in the lungs. However, IL-22 does not play a function in Th2 low asthma. These findings suggest that IL-22 could be targeted as adjuvant therapy for allergic asthma.

## Data Availability

The data can be requested from the corresponding author.
